# Denosumab in patients with osteogenesis imperfecta and a historical control study with alendronate

**DOI:** 10.3389/fendo.2025.1445093

**Published:** 2025-05-27

**Authors:** Yazhao Mei, Shiya Cai, Yunyi Jiang, Yuan Tian, Li Shen, Jiemei Gu, Chun Wang, Zhenlin Zhang, Hao Zhang

**Affiliations:** ^1^ Shanghai Clinical Research Center of Bone Disease, Department of Osteoporosis and Bone Diseases, Shanghai Sixth People’s Hospital Affiliated to Shanghai Jiao Tong University School of Medicine, Shanghai, China; ^2^ Department of Endocrinology, Punan Hospital of Pudong New District, Shanghai, China; ^3^ Clinical Research Center, Shanghai Sixth People’s Hospital Affiliated to Shanghai Jiao Tong University School of Medicine, Shanghai, China

**Keywords:** osteogenesis imperfecta, denosumab, alendronate, hypercalcemia, bone mineral density

## Abstract

**Purpose:**

Optimal dosing of denosumab in osteogenesis imperfecta (OI) remains undefined. This prospective cohort study evaluated the 12-month efficacy and safety of denosumab in OI patients, with a historical control study with alendronate.

**Materials and methods:**

Eight pediatric patients (1 mg/kg every 3 months; ≤60 mg/dose) and ten adults (60 mg every 6 months) received denosumab. Outcomes included lumbar spine (LS) and femoral neck (FN) bone mineral density (BMD), bone turnover markers (BTMs), vertebral compression fractures (assessed via AI-assisted Genant grading [AI_OVF_SH system]), fracture incidence, height velocity and adverse events. Historical controls (n=25 alendronate-treated OI patients) were analyzed for comparative efficacy. Sensitivity analyses excluded female pediatric participants (n=4) and peri-/post-menopausal adults (n=4) to assess hormonal confounding.

**Results:**

Pediatric denosumab recipients exhibited significant LS-BMD (+30.3%, *P*<0.001) and FN-BMD gains (+38.7%, *P*=0.001) versus baseline, whereas adults showed non-significant increases (LS: +2.6%, *P*=0.100; FN: +4.4%, *P*=0.051). Sensitivity analyses revealed attenuated BTMs suppression in adults after excluding peri-/post-menopausal women (only ALP decreased by 27.9%, *P*=0.028). Rebound hypercalcemia occurred in 62.5% (5/8) of children, peaking at 2.93 mmol/L. Compared to alendronate, denosumab demonstrated comparable BMD improvements and fracture reduction (*P*>0.050) but superior pediatric height gain (+5.8% *vs*. +2.5%, *P*=0.004). Vertebral area loss decreased significantly with denosumab (-14.6%, *P*=0.029), unlike alendronate (-8.8%, *P*=0.296). Adverse events were more frequent with denosumab in children (hypercalcemia: 62.5% *vs*. 0%, *P*=0.002).

**Conclusion:**

Denosumab demonstrates non-inferior efficacy to alendronate for BMD improvement in OI, with heightened vertebral remodeling and pediatric height gains. However, its overshoot phenomenon in children (rebound hypercalcemia) and hormone-dependent efficacy in adults necessitate risk-stratified use. Age and menopausal status considerations are critical for optimizing denosumab therapy in OI.

**Clinical trial registration:**

https://www.chictr.org.cn/bin/project/edit?pid=184231, identifier ChiCTR2300074207.

## 1 Introduction

Osteogenesis imperfecta (OI), a monogenic disorder of type I collagen metabolism, is characterized by recurrent fragility fractures, low bone mass, and variable extraskeletal manifestations including dentinogenesis imperfecta, blue sclerae, and joint hyperlaxity (1, 2). Current therapeutic strategies for OI, predominantly adapted from osteoporosis treatments, rely heavily on bisphosphonates (BPs) for their anti-resorptive effects (3). While BPs consistently improve bone mineral density (BMD), their efficacy in fracture risk reduction remains equivocal (4).

Denosumab, a monoclonal antibody targeting RANKL, disrupts osteoclastogenesis through competitive inhibition of the RANKL-RANK pathway (5, 6). In postmenopausal osteoporosis, denosumab demonstrates sustained BMD gains without plateau effects, outperforming BPs in long-term outcomes (7, 8). However, its transient pharmacodynamics—marked by rapid bone turnover markers (BTMs) suppression followed by rebound hyperabsorption upon discontinuation—raise unique safety concerns, particularly in pediatric populations with inherently elevated bone turnover (9, 10).

Emerging evidence supports denosumab’s utility in OI (11–17), but optimal dosing regimens and comparative efficacy against alendronate remain undefined. This 12-month prospective cohort study evaluates denosumab’s safety and efficacy in pediatric and adult OI patients, utilizing a historical alendronate control group to inform clinical decision-making.

## 2 Materials and methods

### 2.1 Study population

Inclusion required confirmed OI diagnosis per Sillence criteria (18): 1) With fracture family history: ≥1 fragility fracture + lumbar/hip BMD Z-score ≤-1; 2) Without fracture family history: ≥1 fragility fracture + ≥1 extraskeletal feature (blue sclerae, dentinogenesis imperfecta, hearing loss, ligamentous laxity). Exclusion criteria encompassed other metabolic or hereditary bone diseases (hyper/hypothyroidism, Paget’s disease, hypophosphatemic rickets/osteomalacia et al.), chronic organ dysfunction, malignancy, glucocorticoid use, pregnancy, or lactation.

### 2.2 Study design

Eligible participants were classified into pediatric (<18 years) or adult groups. A prospective cohort of OI patients was established at the Department of Osteoporosis and Bone Diseases of Shanghai Sixth People’s Hospital Affiliated to Shanghai Jiao Tong University School of Medicine, with pediatric and adult subgroups. Historical controls (alendronate-treated patients with ≥12 months of follow-up) were selected from institutional databases using identical inclusion/exclusion criteria as the denosumab cohort.

Pediatric denosumab (Prolia^®^, Amgen Inc., Thousand Oaks, CA) dosing was 1 mg/kg subcutaneously every 3 months (max 60 mg/dose), while adults received 60 mg every 6 months. Alendronate (Fosamax, Merck Sharp & Dohme, USA) was administered at 70 mg/week. All participants received calcium (300–600 mg/day) and vitamin D (≥400 IU/day).

The primary efficacy endpoints were the changes in BMD and serum levels of BTMs during treatment. Secondary endpoints were the incidence of new fractures, area loss of vertebra and height velocity.

### 2.3 Measurements

Anthropometric measurements (height, weight) were obtained annually. For non-ambulatory patients, supine length was recorded; limb length discrepancies were addressed by measuring the longer extremity. Pediatric heights were converted to SDS using Chinese reference data (18). The body mass index (BMI) was calculated as weight/height² (kg/m²).

Lumbar spine (LS) and femoral neck (FN) BMD were assessed via dual-energy X-ray absorptiometry (DXA; Lunar/Hologic) at baseline and 12 months (19). Daily phantom calibrations ensured quality control (CV: Lunar LS 1.39%, FN 2.22%; Hologic LS 0.9%, FN 0.08%). Scans with surgical implants or deformities were excluded. All measurements used consistent operators/devices, with Z-scores derived from age-sex references (20–22).

Thoracolumbar radiographs (T4-L4) were analyzed using the AI_OVF_SH system, which demonstrated 96.85% fracture detection accuracy (23). Vertebral compression fractures were graded by area loss: Grade 1 (10-20%), Grade 2 (20-40%), Grade 3 (>40%). Scoliosis was defined as Cobb angle >10° (24).

Fasting morning blood samples collected every 3–6 months were analyzed on Hitachi 7600-020 (calcium(Ca, reference range 2.08-2.60mmol/L), phosphorus (P)) and Roche Cobas 6000 (C-terminal telopeptide of type 1 collagen (CTX), osteocalcin (OC), 25-hydroxyvitamin D (25OHD, reference range ≥20 ng/mL), and intact parathyroid hormone (PTH, reference range 15.00-65.00 pg/mL)) platforms. P, CTX, and OC were matched to age adapted reference data (25, 26). Rebound hypercalcemia was defined as serum calcium >2.60 mmol/L without secondary causes.

### 2.4 Treatment safety assessment

Adverse events (AEs) were monitored at each visit, including incident fractures. Serious AEs (SAEs) - cellulitis/erysipelas, osteonecrosis of the jaw, atypical femoral fractures, delayed fracture healing, and cardiac disorders - required clinical and radiographic confirmation. Other AEs (hypocalcemia, hypophosphatemia, hypercalcemia, arthralgia, myalgia) were assessed via clinical and biochemical evaluation.

### 2.5 Statistical analysis

Statistical analyses were performed using SPSS 26.0. Continuous variables were assessed for normality via Kolmogorov-Smirnov test and homogeneity of variance via Levene’s test. Normally distributed continuous variables were expressed as mean ± standard deviation (SD), while non-normally distributed measures were expressed as median (Interquartile range, IQR); categorical variables were expressed as frequencies (%). An independent sample t-test was used for group comparisons of normally distributed data, nonnormally distributed data were compared between groups using nonparametric tests, and rates were compared using chi-square test and Fisher exact test. Within-group comparisons used paired t-tests or Wilcoxon signed-rank tests. Analysis of covariance (ANCOVA) adjusted for age in between-drug analyses. Analyses included all participants receiving ≥1 denosumab dose with baseline assessment (modified intention-to-treat principle). Missing data were addressed via the last observation carried forward. Statistical significance was set at α=0.05 (two-tailed).

### 2.6 Ethical statement

This study was registered in the Chinese Clinical Trial Registry (registration number: ChiCTR2300074207). All procedures were conducted in accordance with the ethical standards of the institutional and national research committees and the 2013 revision of the Declaration of Helsinki. The Ethics Committee of Shanghai Sixth People’s Hospital affiliated to Shanghai Jiao Tong University School of Medicine approved the study. Written informed consent was obtained from patients or legal guardians of children younger than 18.

## 3 Results

### 3.1 Baseline characteristics

**Figure 1:**
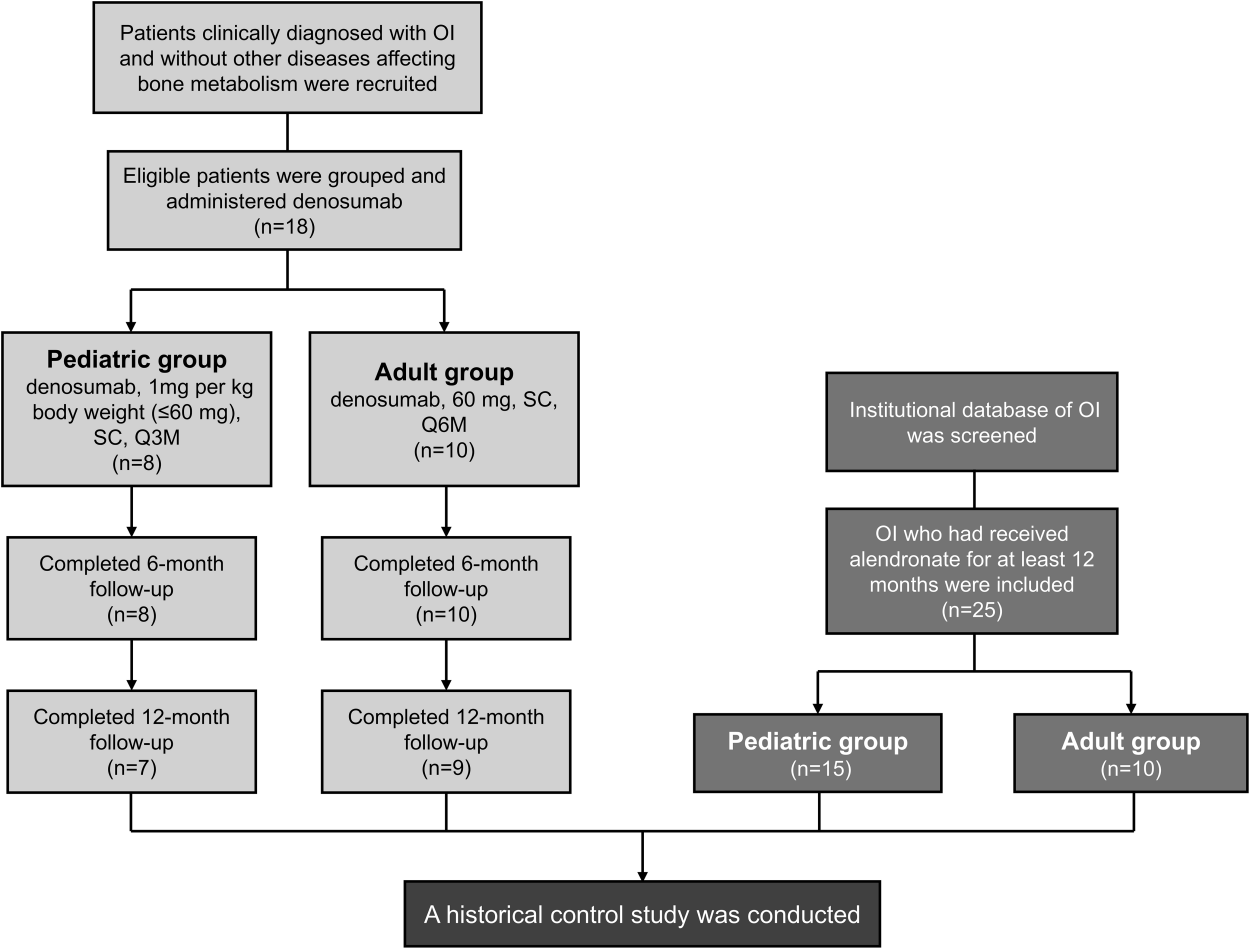
Study flowchart. OI, osteogenesis imperfecta; SC, subcutaneous injection; Q3M, every 3 months; Q6M, every 6 months. Two discontinued at 6 months because of transportation barriers.

The study included 18 denosumab-treated patients (8 pediatrics, 10 adults) and 25 historical alendronate controls (15 pediatrics, 10 adults) (**Figure 1**). Baseline demographics and clinical features were comparable between groups (**Tables 1**, **2**), except for prior treatment history in pediatric patients (*P*=0.002) and age in adults (*P*=0.031). A total of 8 patients had a medication treatment history to increase BMD in denosumab cohort, including 5 children and 3 adults (**Supplementary Figure 1**). Treatment switching rationale included suboptimal BMD response (n=5), new fractures (n=2), or alendronate intolerance (n=1). All alendronate patients were treatment-naïve except one adult (36.9 years) with prior zoledronic acid exposure. All patients underwent genetic testing for OI-associated variants (*COL1A1, COL1A2, IFITM5, WNT1*) and mutations were identified in 40 patients (**Supplementary Table 1**).

**Table 1:** Baseline characteristics of OI children received denosumab or alendronate treatment.

	DEN(N=8)	ALN(N=15)	*P*
Age (years)	9.4 ± 4.4	11.5 ± 4.0	0.257
Sex (boy/girl)	8/0	11/4	0.257
Height (cm)	133.39 ± 28.89	140.97 ± 21.37	0.481
Height Z-score	-0.72 ± 1.53	-1.05 ± 1.20	0.577
Weight (kg)	39.09 ± 25.02	40.33 ± 17.63	0.890
BMI (kg/cm^2^)	19.60 ± 5.67	19.17 ± 3.92	0.831
Fracture rate (times/year)	1.06 ± 0.81	0.79 ± 0.68	0.399
OI type (I/III/IV)	4/1/3/0	8/3/4	1.000
Mutation genes (*COL1A1*/*COL1A2*/*IFITM5*/*WNT1*/unknown)	3/5/0/0/0	10/4/0/1/0	0.253
Treatment history (alendronate/ibandronate/zoledronic acid/none)	4/1/0/3	0/0/0/15	**0.002**
Ca (mmol/L)	2.45 ± 0.08	2.40 ± 0.11	0.277
P (mmol/L)	1.63 ± 0.07	1.50 ± 0.17	0.058
25OHD (ng/ml)	28.59 ± 14.86	28.12 ± 15.74	0.947
PTH (pg/ml)	43.73 ± 18.87	36.23 ± 25.30	0.485
CTX (ng/L)	1196.05 ± 231.48	1133.87 ± 375.41	0.685
ALP (U/L)	341.63 ± 140.65	284.92 ± 147.62	0.403
P1NP (ng/ml)	540.10 ± 315.90	/	/
OC (ng/ml)	112.24 ± 36.48	110.92 ± 76.19	0.965
LS BMD (g/cm^2^)	0.670 ± 0.350	0.602 ± 0.182	0.542
LS Z-score	-1.09 (-1.81, 0.98)	-1.73 (-3.20, -0.10)	0.169
FN BMD (g/cm^2^)	0.609 ± 0.328	0.626 ± 0.219	0.891
FN Z-score	-1.74 (-2.28, -1.20)	-2.35 (-3.36, -0.24)	0.485
Area loss ratio of vertebra	13.41 ± 4.19%	11.78 ± 7.89%	0.345

OI, osteogenesis imperfecta; DEN, denosumab; ALN, alendronate; BMI, body mass index; 25OHD, 25-hydroxyvitamin D; PTH, parathyroid hormone; CTX, C-terminal telopeptide of type 1 collagen; ALP, alkaline phosphatase; P1NP, type I N-terminal propeptide of type 1 procollagen; OC, osteocalcin; BMD, bone mineral density; LS, lumbar spine; FN, femoral neck. Statistical significance was defined as two-tailed P < 0.05, with significant results bolded.

**Table 2:** Baseline characteristics of OI adults received denosumab or alendronate treatment.

	DEN(N=10)	ALN(N=10)	*P*
Age (years)	46.6 ± 14.6	34.3 ± 6.8	**0.031**
Sex (male/female)	1/9	4/6	0.303
Height (cm)	149.28 ± 7.45	154.70 ± 6.52	0.315^*^
Weight (kg)	50.17 ± 7.04	57.32 ± 8.77	0.261^*^
BMI (kg/cm^2^)	22.52 ± 2.73	24.00 ± 3.68	0.400^*^
Fracture rate	0.26 ± 0.22	0.27 ± 0.22	0.395^*^
OI types (I/III/IV/V)	8/0/1/1	9/0/1/0	1.000
Mutation genes (*COL1A1*/*COL1A2*/*IFITM5*/*WNT1*/unknown)	7/1/1/0/1	8/0/0/0/2	1.000
Treatment history (alendronate/ibandronate/zoledronic acid/none)	2/0/1/7	0/0/1/9	0.721
Ca (mmol/L)	2.37 ± 0.06	2.37 ± 0.10	0.696^*^
P (mmol/L)	1.14 ± 0.15	1.02 ± 0.20	0.369^*^
25OHD (ng/ml)	27.18 ± 8.85	23.11 ± 15.42	0.360^*^
PTH (pg/ml)	43.26 ± 10.25	48.42 ± 11.27	0.695^*^
CTX (ng/L)	328.10 ± 261.06	279.47 ± 164.64	0.378^*^
ALP (U/L)	81.70 ± 26.20	77.71 ± 27.08	0.717^*^
P1NP (ng/ml)	32.11 ± 23.39	/	/
OC (ng/ml)	25.98 ± 18.21	21.31 ± 8.72	0.578^*^
LS BMD (g/cm^2^)	0.781 ± 0.085	0.851 ± 0.113	0.749^*^
LS Z-score	-2.11 ± 0.56	-2.09 ± 0.97	0.802^*^
FN BMD (g/cm^2^)	0.655 ± 0.111	0.783 ± 0.096	0.067^*^
FN Z-score	-1.50 ± 0.76	-1.13 ± 0.83	0.162^*^

OI, osteogenesis imperfecta; DEN, denosumab; ALN, alendronate; BMI, body mass index; 25OHD, 25-hydroxyvitamin D; PTH, parathyroid hormone; CTX, C-terminal telopeptide of type 1 collagen; ALP, alkaline phosphatase; P1NP, type I N-terminal propeptide of type 1 procollagen; OC, osteocalcin; BMD, bone mineral density; LS, lumbar spine; FN, femoral neck; *: age is corrected by the covariance analysis. Statistical significance was defined as two-tailed P < 0.05, with significant results bolded.

### 3.2 Primary outcomes

#### 3.2.1 Denosumab cohort

7/8 pediatric (87.5%) and 9/10 adult (90%) patients completed 12-month follow-up. Two discontinued at 6 months because of transportation barriers.

**Figure 2:**
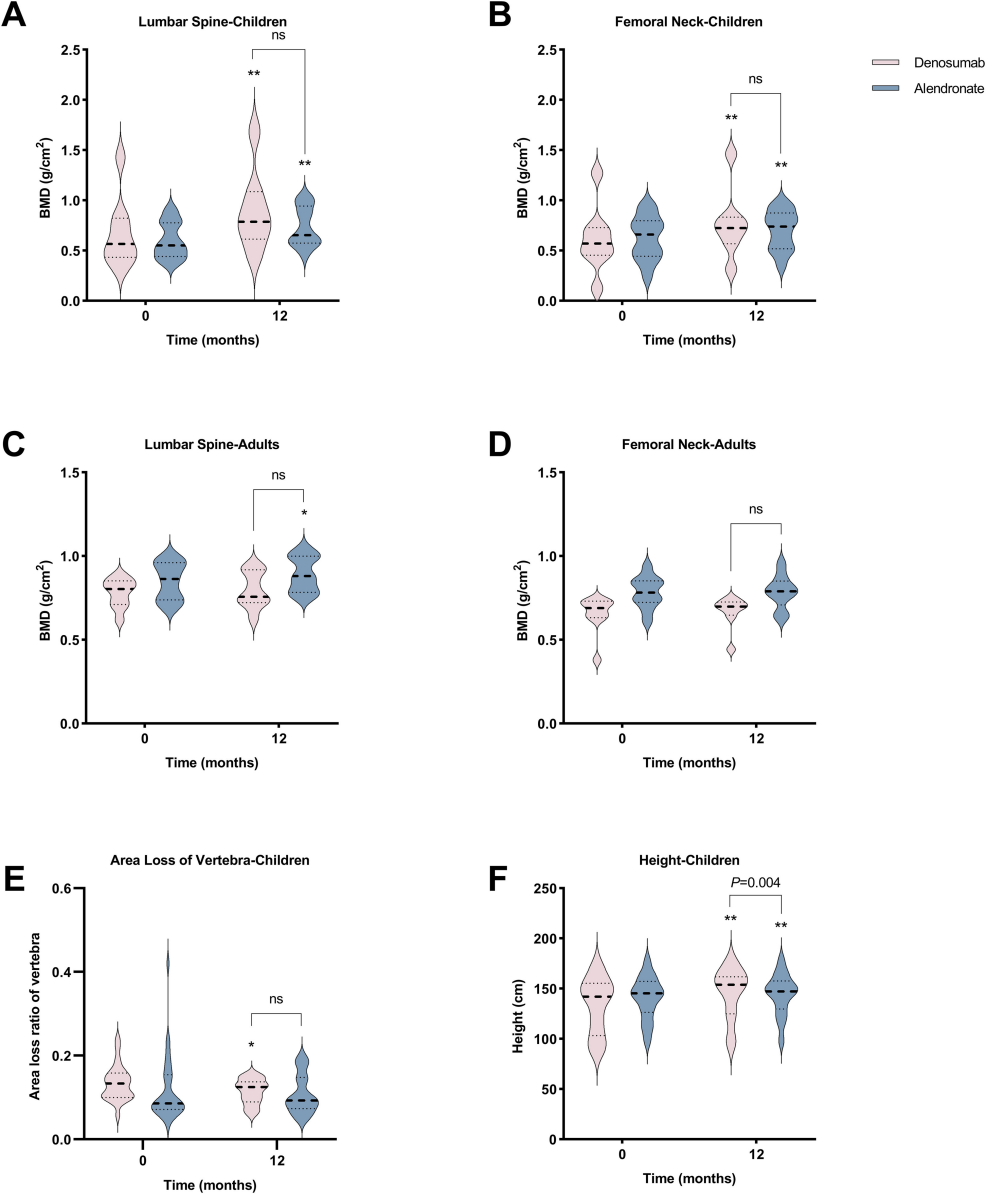
Comparative effects of denosumab and alendronate on bone parameters. **(A-D)** Longitudinal changes in lumbar spine (LS) and femoral neck (FN) bone mineral density BMD in pediatric and adult cohorts. **(E)** Vertebral area loss ratio progression in pediatric patients. **(F)** Height velocity in pediatric cohort. Data are presented as mean ± SD. *P<0.05, **P<0.01 *vs* baseline; ns, no significant intergroup differences (denosumab *vs* alendronate).

**Figure 3:**
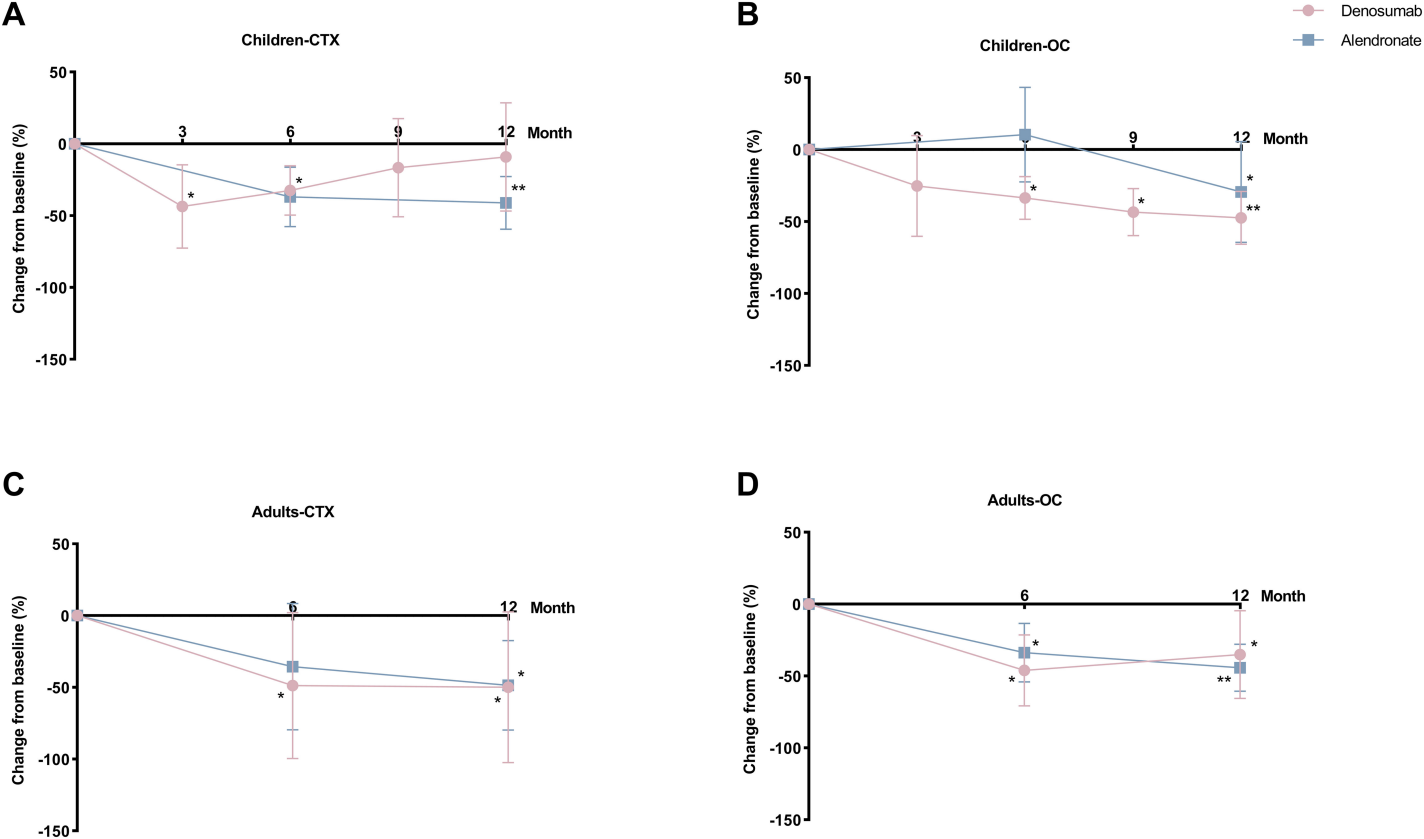
Percent changes in bone turnover markers (BTMs) with denosumab and alendronate. **(A, B)** Serum C-terminal telopeptide of type 1 collagen (CTX) and osteocalcin (OC) dynamics in pediatric patients. **(C, D)** Corresponding BTMs changes in adult patients. Data expressed as percentage change from baseline. *P<0.05, **P<0.01 *vs* baseline.

After 12 months of denosumab therapy, significant BMD increases at LS (+30.3%, *P*<0.001) and FN (+38.7%, *P*=0.001) were observed in pediatric group (**Figures 2A, B**). CTX levels showed transient suppression (43.5% reduction at 3 months and 32.4% at 6 months) with a rebound to baseline by 9 months (**Figure 3A**). OC levels exhibited persistent suppression in the whole period (**Figure 3B**). In the adult group, denosumab treatment led to modest BMD gains (LS: +2.6%, *P*=0.100; FN: +4.4%, *P*=0.051; **Figures 2C, D**) with sustained CTX suppression (48.7% reduction at 6 months and 49.9% at 12 months, *P*<0.050; **Figure 3C**).

#### 3.2.2 Alendronate cohort

In the pediatric group, 12-month alendronate treatment significantly increased BMD (LS: +24.0%, *P*<0.001; FN: +15.7%, *P*<0.001; **Figures 2A, B**), with CTX levels declining steadily (*P*<0.050; **Figure 3A**). In adult alendronate recipients, a 5.1% LS BMD gain (P=0.010) and 1.6% FN BMD improvement (P=0.051; **Figures 2C, D**) were accompanied by sustained reductions in serum CTX and OC levels throughout the 12-month treatment period (**Figures 3C, D**).

#### 3.2.3 Comparative analysis between denosumab and alendronate

No significant differences in BMD changes or BTMs suppression were observed between denosumab and alendronate groups (**Figures 2**, **3**).

### 3.3 Secondary outcomes

Two pediatric denosumab recipients experienced new fractures (rate: 0.83/year), while no fractures occurred in adults. Alendronate-treated pediatric patients had a similar fracture rate (0.25/year, *P*>0.050). Denosumab significantly reduced vertebral area loss in pediatric patients (from 13.4% to 11.5%, *P*=0.029) (**Figure 2E**), with two cases showing fracture remodeling (**Supplementary Figure 2**). Alendronate also promoted remodeling but without significant area loss reduction (from 11.8% to 10.8%, *P*=0.296). Denosumab-treated pediatric patients showed greater height increase compared to alendronate recipients (+5.8% *vs* +2.5%, *P*=0.004) (**Figure 2F**).

### 3.4 Treatment safety assessment

**Figure 4:**
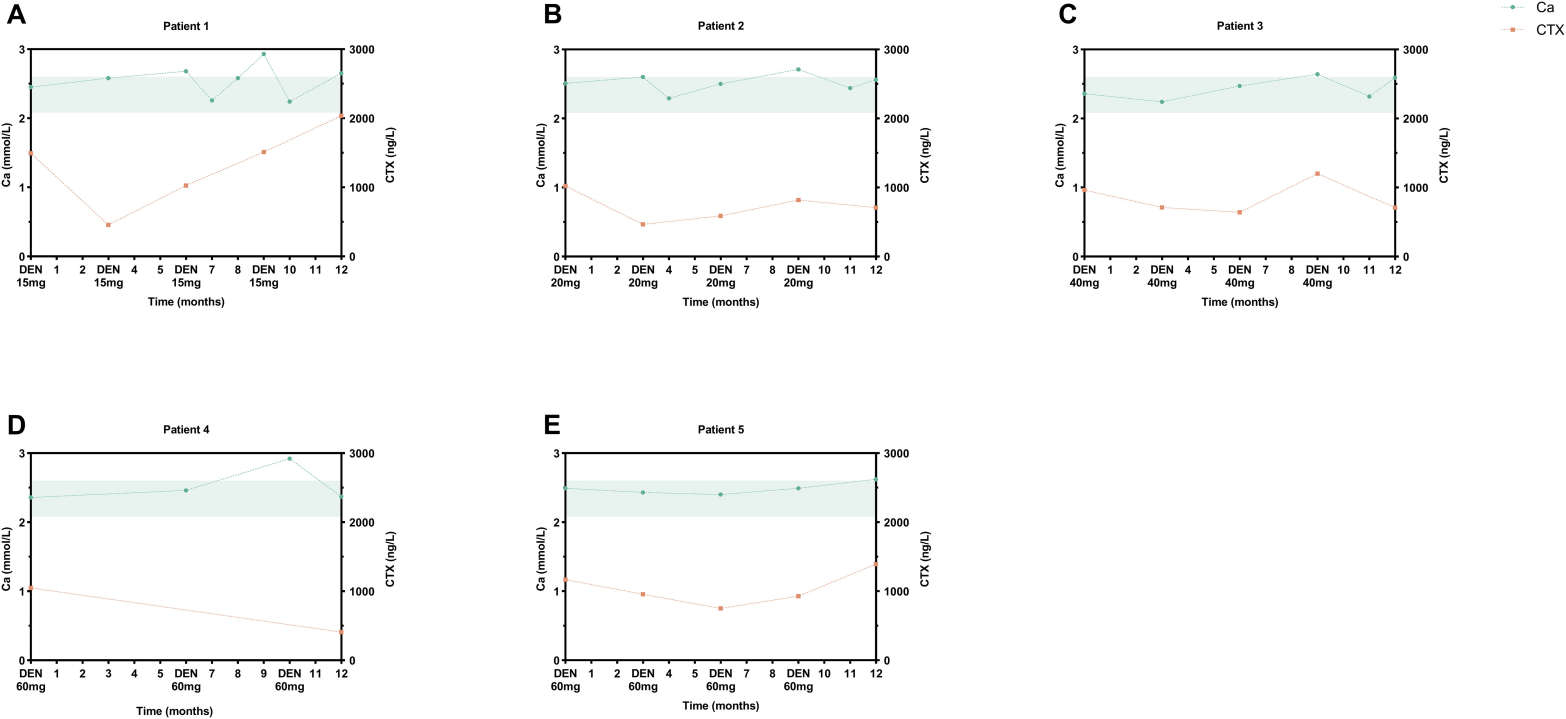
Hypercalcemia events in pediatric denosumab recipients. Serial serum calcium (reference range: 2.08-2.60 mmol/L, shaded green) and CTX profiles of five cases: **(A)** 2-year-old male (15 mg), transient hypercalcemia at M6/9/12; **(B)** 5.9-year-old male (20 mg), M9 elevation; **(C)** 12.3-year-old male (40 mg), M10 event; **(D)** 13.9-year-old male (60 mg), M10 episode; **(E)** 11-year-old male (60 mg), M12 occurrence. DEN, denosumab.

Treatment-emergent AEs are detailed in **Table 3**. No SAEs occurred in either cohort during the 12-month study period. In denosumab cohort, five children (5/16, 31.3%) developed mild hypercalcemia (peak serum Ca: 2.93 mmol/L), consistently occurring 3 months post-dosing (**Figure 4**). These episodes coincided with rebound increases in CTX to near-baseline levels. Compared to alendronate, denosumab pediatric recipients demonstrated significantly higher risks of hypercalcemia (62.5% *vs*. 13.3%; *P*<0.002), secondary hyperparathyroidism (62.5% *vs*. 13.3%; *P*<0.026), and musculoskeletal symptoms (37.5% *vs*. 0%; *P*<0.008). Both therapies exhibited favorable safety profiles in adults.

**Table 3:** Adverse events in denosumab and alendronate.

A. In OI children			
	DEN (n=8)	ALN (n=15)	*P*
Serious adverse events			
cellulitis or erysipelas	0(0/8)	0(0/15)	1.000
osteonecrosis of the jaw	0(0/8)	0(0/15)	1.000
atypical femoral fracture	0(0/8)	0(0/15)	1.000
delayed fracture healing	0(0/8)	0(0/15)	1.000
cardia disorders	0(0/8)	0(0/15)	1.000
Other relevant adverse events			
hypocalcemia	1(1/8)	0(0/15)	0.348
hypercalcemia	5(5/8)	0(0/15)	**0.002**
hypophosphatemia	1(1/8)	0(0/15)	0.348
Secondary hyperparathyroidism	5(5/8)	2(2/15)	**0.026**
Secondary hypoparathyroidism	2(2/8)	0(0/15)	0.111
arthralgia	4(4/8)	0(0/15)	**0.008**
muscle pain	4(4/8)	0(0/15)	**0.008**
**Total**	5(5/8)	2(2/15)	**0.026**
B. In OI adults			
	DEN (N=10)	ALN (N=10)	*P*
Serious adverse events			
cellulitis or erysipelas	0(0/10)	0(0/10)	1.000
osteonecrosis of the jaw	0(0/10)	0(0/10)	1.000
atypical femoral fracture	0(0/10)	0(0/10)	1.000
delayed fracture healing	0(0/10)	0(0/10)	1.000
cardia disorders	0(0/10)	0(0/10)	1.000
Other relevant adverse events			
hypocalcemia	1(1/10)	0(0/10)	1.000
hypercalcemia	0(0/10)	0(0/10)	1.000
hypophosphatemia	0(0/10)	0(0/10)	1.000
Secondary hyperparathyroidism	3(3/10)	0(0/10)	0.211
Secondary hypoparathyroidism	0(0/10)	0(0/10)	1.000
arthralgia	0(0/10)	0(0/10)	1.000
muscle pain	0(0/10)	0(0/10)	1.000
**Total**	3(3/10)	0(0/10)	0.211

OI, osteogenesis imperfecta; DEN, denosumab; ALN, alendronate. Statistical significance was defined as two-tailed P < 0.05, with significant results bolded.

### 3.5 Sensitivity analysis

Notably, the study cohort exhibited marked sex disparities: all pediatric denosumab recipients were male (8), while 90% (9/10) of adult denosumab users were female, including three peri-/post-menopausal individuals. To address potential confounding from hormonal status and sex-specific effects, sensitivity analyses were performed: 1) Exclusion of female pediatric participants: Removal of 4 girls from the alendronate cohort (**Supplementary Table 2**). 2) Exclusion of peri-/post-menopausal adults: Omission of 3 denosumab-treated and 1 alendronate-treated women (**Supplementary Table 3**). After excluding peri-/post-menopausal females, denosumab’s effect on BTMs was attenuated, with only alkaline phosphatase (ALP) demonstrating a significant 27.9% reduction post-treatment (*P*=0.028 *vs*. baseline). Results remained stable in the denosumab pediatric subgroup. Comparative efficacy between denosumab and alendronate remained robust across all sensitivity analyses (*P*>0.050).

## 4 Discussion

This study provides the first head-to-head comparison of denosumab and alendronate in OI. Pediatric denosumab recipients exhibited marked BMD gains and vertebral remodeling, consistent with prior reports (11–17). However, the high incidence of rebound hypercalcemia (62.5%) underscores metabolic instability in children.

Since Semler et al. (2012) first reported safe BTMs suppression in four pediatric OI-VI patients with quarterly 1 mg/kg denosumab (27), subsequent studies (11–15, 28, 29) have confirmed its BMD-enhancing effects but identified pediatric-specific rebound risks. Our protocol adopted weight-based pediatric dosing (1 mg/kg/Q3M, ≤60 mg) versus fixed adult regimens (60 mg/Q6M). At 12 months, pediatric LS/FN BMD increased significantly versus non-significant adult changes, potentially attributable to ongoing skeletal growth (30). Pharmacodynamic analyses reveal accelerated drug metabolism in children: BTMs rebound to baseline within 6–8 weeks (27, 31), correlating with 30-50% higher baseline BTMs versus adults (32). Notably, 31% (13/42) pediatric patients in a 6-month interval trial developed rebound hypercalcemia (17), while our 3-month regimen still showed pre-dose CTX recovery. These findings suggest current intervals may inadequately suppress resorption, warranting exploration of compressed schedules (e.g., ≤10 weeks) as proposed by Semler (29).

Fracture patterns in denosumab-treated OI patients demonstrate age-related disparities. While adult fracture rates decreased versus baseline, two pediatric cases developed new fractures - consistent with established epidemiology showing fracture burden predominantly in pediatric populations (33). This inherent limitation (small sample, non-randomized design) precludes definitive fracture risk assessment in our cohort.

Overshoot phenomena remain a critical safety concern. Pediatric patients exhibited CTX rebound by month 3 post-dose, with 5 cases developing mild hypercalcemia (2 with hypoparathyroidism, 3 with musculoskeletal symptoms). A review of denosumab in pediatric bone diseases summarized that 45 children treated with denosumab for various conditions developed “severe hypercalcemia” in 5 cases after discontinuation, and mild or moderate asymptomatic hypercalcemia was reported in 6 of the 18 children with OI treated with denosumab (34). Younger age (<5 years) and elevated resorption markers correlate with risk (17). Mechanistically, rapid CTX rebound drives osteoclast-mediated calcium release (35), occurring earlier in children (3 *vs*. 6–10 months in adults) due to accelerated bone turnover (36). These findings align with MHRA’s 2022 warning against pediatric denosumab use (37).

Comparative analyses show denosumab’s BMD superiority over BPs in osteoporosis (38–40), though fracture reduction equivalence persists. Our historical controls revealed comparable BMD and BTMs responses between denosumab and alendronate, but denosumab demonstrated superior vertebral remodeling and greater height velocity despite preclinical growth concerns (34). Notably, denosumab carried higher hypercalcemia risk, attributable to its potent resorption inhibition (36). While both agents enable vertebral reshaping in OI (17, 41–43), denosumab’s unique pharmacodynamics warrant careful risk-benefit evaluation in pediatric use.

Our sensitivity analyses revealed that the BTMs-lowering effect of denosumab in adults was predominantly driven by peri-/post-menopausal women. This aligns with the known acceleration of bone turnover during estrogen withdrawal, which may amplify denosumab’s anti-resorptive potency (44). The attenuation of BTMs suppression after excluding these individuals underscores the importance of hormonal milieu in modulating therapeutic responses (45). Our findings highlight the need for stratified therapeutic approaches: in postmenopausal OI women, denosumab may offer enhanced anti-resorptive efficacy, whereas pediatric patients may require closer monitoring of BTMs to detect early rebound effects.

This pioneering real-world analysis provides the first comparative assessment of denosumab versus alendronate in OI patients across pediatric and adult populations. Key limitations merit consideration: 1) 12-month duration precluded evaluation of long-term risks (e.g., ONJ, atypical fractures); 2) Underpowered sample limits fracture risk assessment validity, particularly in pediatric subgroups; 3) Prior BP exposure in 35% of denosumab recipients may confound therapeutic comparisons; 4) Absence of serial renal ultrasounds prevented hypercalcemia-related calcification monitoring; 5) Historical control design introduces residual confounding (e.g., age/sex disparities)., however, such limitations are unavoidable in rare disease contexts.

## 5 Conclusions

Denosumab demonstrates potential for improving BMD and vertebral structure in OI, particularly in children. However, its association with rebound hypercalcemia and metabolic instability necessitates caution. Alendronate remains a safer alternative for pediatric OI until further evidence establishes denosumab’s risk-benefit balance. Clinicians should prioritize individualized treatment plans considering age and menopausal status and vigilant monitoring for patients on denosumab.

## Data Availability

The raw data supporting the conclusions of this article will be made available by the authors, without undue reservation.

## Supplementary material

Supplementary Figure 1 Prior treatment history of denosumab-treated OI patients. Pediatric cohort: P1 (14M) - 4-year alendronate (10-14y); P2 (11M) - single ibandronate dose (9y); P3 (11M) - 3-month alendronate (10y) with 6-month gap; P5 (12M) - ongoing alendronate (10y-). Adult cohort: P6 (68F) - 4-year alendronate (64-68y); P7 (33F) - 4-month alendronate; P8 (40F) - sequential alendronate (2y) and zoledronic acid (2y) (36-40y).

Supplementary Figure 2 Radiographic evidence of vertebral remodeling. **(A)** Thoracolumbar series (13M): Pre-treatment *vs* 12-month DEN. **(B)** Lumbar progression (13.9M): Baseline to 4-year ALN to 1-year DEN. **(C)** 6-month ALN effects (12.7M). **(D)** 12-month ALN outcomes (10.9M). **(E)** Lower extremity cortical changes (15M): 24-month ALN comparison. Orange arrows denote vertebral remodeling sites/cortical thickening.
