# Supplementary material for: Denosumab in patients with osteogenesis imperfecta and a historical control study with alendronate
Source: Front Endocrinol (Lausanne). 2025 May 27;16:1445093. doi: 10.3389/fendo.2025.1445093 (PMC12148890; doi:10.3389/fendo.2025.1445093)
Supplement: Supplementary file 1 [file Table1.docx]

**Supplementary Table 1 Genetic Variants identified in the study**

| Group | Sex | Age (Years) | Gene | cDNA |
| --- | --- | --- | --- | --- |
| Denosumab | Male | 13.9 | *COL1A2* | c.1009G>A |
|  | Male | 11.4 | *COL1A2* | c.847G>A |
|  | Male | 2 | *COL1A1* | c.285C>A |
|  | Male | 11 | *COL1A2* | c.1504G>A |
|  | Male | 13.4 | *COL1A1* | c.3655G>A |
|  | Male | 5.3 | *COL1A1* | c.268G>T |
|  | Male | 12.3 | *COL1A2* | c.2314G>A |
|  | Male | 5.9 | *COL1A2* | c.2314G>A |
|  | Female | 68.2 | *COL1A1* | c.3467dupA |
|  | Female | 34 | *IFITM5* | c.-14C>T |
|  | Female | 38 | *COL1A1* | c.697-2_697-1delAG |
|  | Male | 65.2 | *COL1A1* | c.697-2_697-1delAG |
|  | Female | 62 | *COL1A1* | c.268G>T |
|  | Female | 53 | *COL1A1* | c.268G>T |
|  | Female | 40 | *COL1A1* | c.3076C>T |
|  | Female | 26.2 | *COL1A1* | c.3008delC |
|  | Female | 44 | *COL1A2* | c.3583T>C |
| Alendronate | Male | 14 | *COL1A1* | c.3467dupA |
|  | Male | 15 | *COL1A2* | c.2035G>A |
|  | Female | 5 | *COL1A1* | c.1148G>C |
|  | Female | 15.6 | *COL1A2* | c.2827G>A |
|  | Male | 10.9 | *WNT1* | c.500dupG; c.506G>A |
|  | Female | 3.9 | *COL1A1* | c.3226G>A |
|  | Male | 10.6 | *COL1A1* | c.3540delC |
|  | Male | 8 | *COL1A1* | c.2410G>T |
|  | Male | 14 | *COL1A1* | c.91C>T |
|  | Male | 9.8 | *COL1A1* | c.569delC |
|  | Male | 14.3 | *COL1A1* | c.1787G>C |
|  | Male | 7.5 | *COL1A2* | c.812G>A |
|  | Female | 17.8 | *COL1A2* | c.3583T>C |
|  | Male | 12.7 | *COL1A1* | c.1085G>C |
|  | Male | 13.7 | *COL1A1* | c.3040C>T |
|  | Female | 37.7 | *COL1A1* | c.588+2T>A |
|  | Female | 38 | *COL1A1* | c.769G>A |
|  | Male | 22 | *COL1A1* | c.299-1G>A |
|  | Male | 27.4 | *COL1A1* | c.3235G>A |
|  | Female | 30.5 | *COL1A1* | c.1821+1G>A |
|  | Male | 33 | *COL1A1* | c.977G>A |
|  | Female | 36.9 | *COL1A1* | c.268G>T |
|  | Female | 46.8 | *COL1A1* | c.1127delC |

**Supplementary Table 2** Comparisons before and after treatment in OI boys

|  | DEN | | | ALN | |  | P^a^ | P^b^ |
| --- | --- | --- | --- | --- | --- | --- | --- | --- |
|  | Baseline (N=8) | After 12-month treatment (N=7) | P | Baseline (N=11) | After 12-month treatment (N=11) | P |  |  |
| Age (years) | 9.4±4.4 | 11.0±4.4 | **＜0.001** | 11.9±2.6 | 12.8±2.6 | **＜0.001** | 0.187 | / |
| Height (cm) | 133.39±28.89 | 145.86±26.46 | **＜0.001** | 146.22±17.77 | 150.16±16.26 | **＜0.001** | 0.247 | **0.014** |
| Weight (kg) | 39.09±25.02 | 50.13±31.56 | **0.034** | 42.36±17.07 | 45.91±15.89 | **0.012** | 0.738 | 0.555 |
| BMI (kg/cm^2^) | 19.60±5.67 | 21.35±7.53 | 0.277 | 18.93±3.56 | 19.78±3.46 | 0.170 | 0.755 | 0.736 |
| Fracture rate | 1.06±0.81 | 0.83±0.50 | 0.094 | 0.83±0.77 | 0.69±0.52 | 0.153 | 0.542 | 0.290 |
| New fracture rate | / | 0.00 (0.25) | / | / | 0.00 (0.00) | / | / | 0.840 |
| Ca (mmol/L) | 2.45±0.08 | 2.50±0.13 | 0.139 | 2.39±0.12 | 2.36±0.14 | 0.724 | 0.296 | 0.309 |
| P(mmol/L) | 1.63±0.07 | 1.71±0.35 | 0.525 | 1.45±0.14 | 1.34±0.18 | 0.197 | **0.030** | 0.223 |
| 25OHD (ng/ml) | 28.59±14.86 | 23.94±9.87 | 0.340 | 24.11±7.18 | 27.27±6.87 | 0.150 | 0.895 | 0.108 |
| PTH (pg/ml) | 43.73±18.87 | 38.49±29.43 | 0.240 | 43.87±29.41 | 58.50±30.07 | 0.312 | 0.808 | 0.053 |
| CTX (ng/L) | 1196.05±231.48 | 1133.34±577.31 | 0.653 | 1188.70±474.98 | 694.85±325.95 | **0.029** | 0.981 | 0.113 |
| ALP (U/L) | 341.63±140.65 | 219.86±127.41 | **0.002** | 348.71±156.78 | 241.86±120.71 | **0.046** | 0.895 | 0.437 |
| P1NP (ng/ml) | 540.10±315.90 | 482.41±373.42 | 0.127 | / | / | / | / | / |
| OC (ng/ml) | 112.24±36.48 | 53.98±21.96 | **0.004** | 154.57±78.63 | 87.48±42.34 | **0.029** | 0.383 | 0.460 |
| LS BMD (g/cm^2^) | 0.670±0.350 | 0.897±0.403 | **＜0.001** | 0.581±0.169 | 0.725±0.190 | **＜0.001** | 0.472 | 0.531 |
| LS Z-score | -0.23±3.21 | 1.23±2.82 | **0.012** | -1.49±1.37 | -0.39±1.67 | **0.002** | 0.208 | 0.422 |
| FN BMD (g/cm^2^) | 0.609±0.328 | 0.766±0.352 | **0.001** | 0.616±0.215 | 0.692±0.198 | **0.001** | 0.959 | 0.206 |
| FN Z-score | -1.71 (2.71) | -0.66 (2.24) | 0.075 | -2.03 (3.78) | -1.19 (3.67) | **0.017** | 0.601 | 0.616 |

OI: osteogenesis imperfecta; DEN: denosumab; ALN: alendronate; BMI: body mass index; 25OHD: 25-hydroxyvitamin D; PTH: parathyroid hormone; CTX: C-terminal telopeptide of type 1 collagen; ALP: alkaline phosphatase; P1NP: type I N-terminal propeptide of type 1 procollagen; OC: osteocalcin; BMD: bone mineral density; LS: lumbar spine; FN: femoral neck; ^a^: baseline DEN VS baseline ALN; ^b^: DEN% VS ALN%. Statistical significance was defined as two-tailed *P* < 0.05, with significant results bolded.

**Supplementary Table 3** Comparisons before and after treatment in OI adults without peri- or post- menopausal women

|  | DEN | | | ALN | |  | P^a^ | P^b^ |
| --- | --- | --- | --- | --- | --- | --- | --- | --- |
|  | Baseline (N=7) | After 12-month treatment (N=7) | P | Baseline (N=9) | After 12-month treatment (N=9) | P |  |  |
| Age (years) | 40.4±12.2 | 41.4±12.2 | **＜0.001** | 32.9±5.5 | 33.9±5.5 | **＜0.001** | 0.119 | / |
| Sex (male/female) | 1/6 | 1/6 | / | 4/5 | 4/5 | / | 0.308 | / |
| Height (cm) | 147.39±8.13 | 147.33±7.45 | 0.903 | 156.01±5.33 | 156.33±5.67 | 0.472 | **0.023** | 0.597 |
| Weight (kg) | 47.86±5.90 | 48.67±5.57 | 0.227 | 57.91±9.08 | 58.53±10.06 | 0.620 | **0.024** | 0.764 |
| BMI (kg/cm^2^) | 22.12±2.95 | 22.47±2.55 | 0.202 | 23.83±3.86 | 23.95±4.02 | 0.833 | 0.347 | 0.347 |
| Fracture rate | 0.34±0.22 | 0.32±0.21 | 0.058 | 0.29±0.23 | 0.27±0.22 | **0.033** | 0.669 | 0.767 |
| New fracture rate | / | 0.00±0.00 | **/** | / | 0.00±0.00 | **/** | / | / |
| Ca (mmol/L) | 2.36±0.07 | 2.34±0.14 | 0.750 | 2.36±0.10 | 2.30±0.09 | 0.062 | 0.929 | 0.413 |
| P (mmol/L) | 1.16±0.12 | 1.15±0.16 | 0.732 | 1.00±0.21 | 0.96±0.13 | 0.580 | 0.096 | 0.875 |
| 25OHD (ng/ml) | 22.54±4.20 | 27.73±7.96 | 0.178 | 24.76±15.87 | 31.47±16.13 | 0.150 | 0.727 | 0.920 |
| PTH (pg/ml) | 43.59±9.21 | 40.87±16.96 | 0.690 | 48.71±12.14 | 46.02±12.20 | 0.131 | 0.391 | 0.545 |
| ALP (U/L) | 80.43±27.07 | 51.00±10.45 | **0.028** | 78.33±29.62 | 59.00±23.45 | **0.003** | 0.896 | 0.619 |
| CTX (ng/L) | 311.01±279.01 | 97.11±62.18 | 0.053 | 274.10±177.08 | 116.06±41.27 | **0.046** | 0.773 | 0.365 |
| P1NP (ng/ml) | 36.60±26.36 | 18.06±11.49 | 0.071 | / | / | / | / | / |
| OC (ng/ml) | 26.40±20.39 | 11.26±3.01 | 0.065 | 19.15±6.72 | 11.89±3.10 | **0.009** | 0.389 | 0.991 |
| LS BMD (g/cm^2^) | 0.804±0.074 | 0.829±0.097 | 0.124 | 0.866±0.109 | 0.907±0.101 | **0.010** | 0.222 | 0.465 |
| LS Z-score | -2.33±0.35 | -2.10±0.46 | 0.084 | -1.97±0.94 | -1.60±0.86 | **0.004** | 0.311 | 0.124 |
| FN BMD (g/cm^2^) | 0.682±0.049 | 0.701±0.040 | 0.144 | 0.788±0.100 | 0.789±0.107 | 0.356 | **0.033** | 0.423 |
| FN Z-score | -1.60±0.43 | -1.43±0.25 | 0.144 | -1.16±0.87 | -1.10±0.91 | 0.109 | 0.214 | 0.374 |

OI: osteogenesis imperfecta; DEN: denosumab; ALN: alendronate; BMI: body mass index; 25OHD: 25-hydroxyvitamin D; PTH: parathyroid hormone; CTX: C-terminal telopeptide of type 1 collagen; ALP: alkaline phosphatase; P1NP: type I N-terminal propeptide of type 1 procollagen; OC: osteocalcin; BMD: bone mineral density; LS: lumbar spine; FN: femoral neck; ^a^: baseline DEN VS baseline ALN; ^b^: DEN% VS ALN%. Statistical significance was defined as two-tailed *P* < 0.05, with significant results bolded.
