# Supplementary figures and images for: Denosumab in patients with osteogenesis imperfecta and a historical control study with alendronate
Source: Front Endocrinol (Lausanne). 2025 May 27;16:1445093. doi: 10.3389/fendo.2025.1445093 (PMC12148890; doi:10.3389/fendo.2025.1445093)

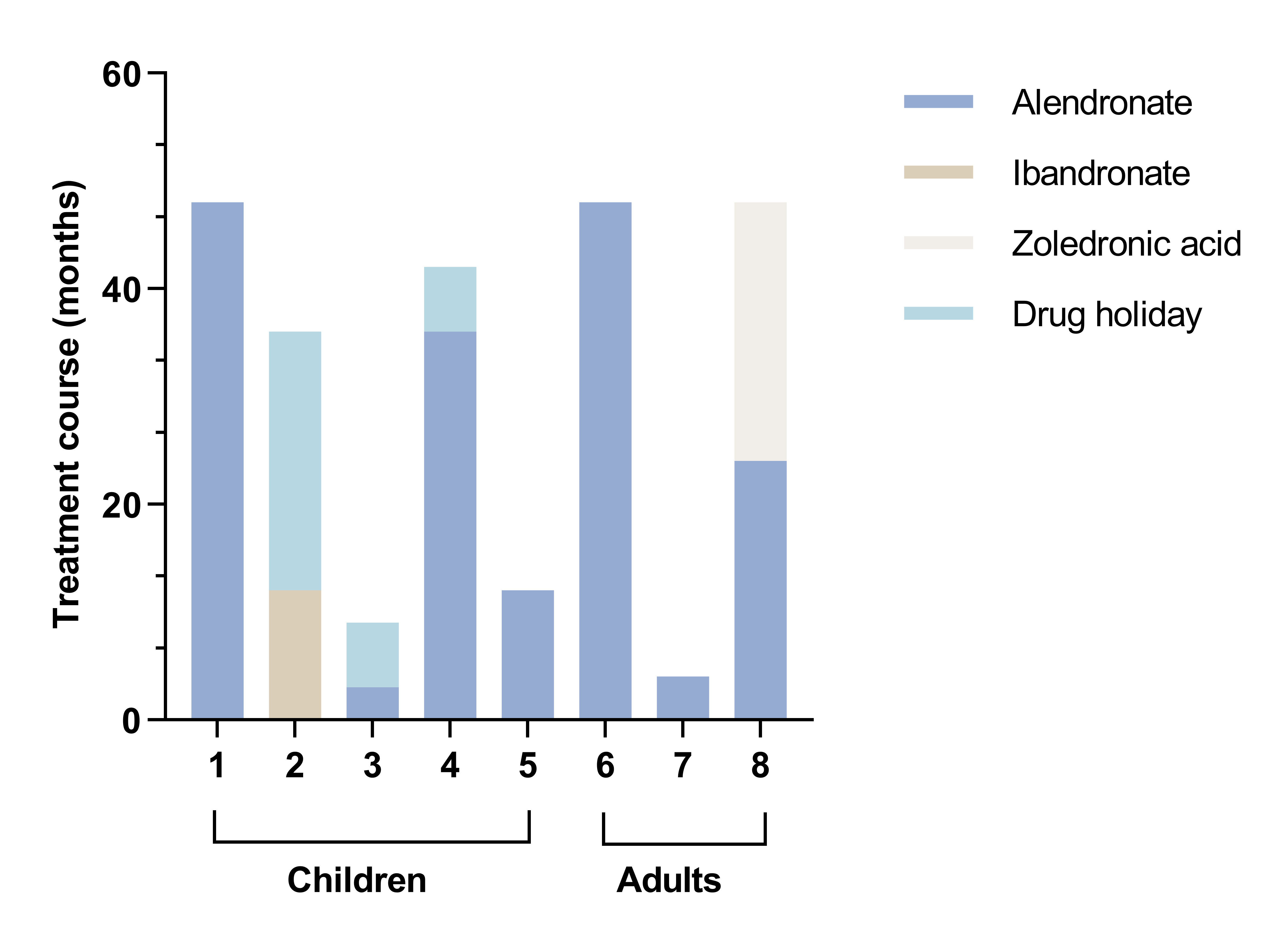

Supplement: Supplementary Figure 1 — Prior treatment history of denosumab-treated OI patients. Pediatric cohort: P1 (14M) - 4-year alendronate (10-14y); P2 (11M) - single ibandronate dose (9y); P3 (11M) - 3-month alendronate (10y) with 6-month gap; P5 (12M) - ongoing alendronate (10y-). Adult cohort: P6 (68F) - 4-year alendronate (64-68y); P7 (33F) - 4-month alendronate; P8 (40F) - sequential alendronate (2y) and zoledronic acid (2y) (36-40y). [file Image1.tif]

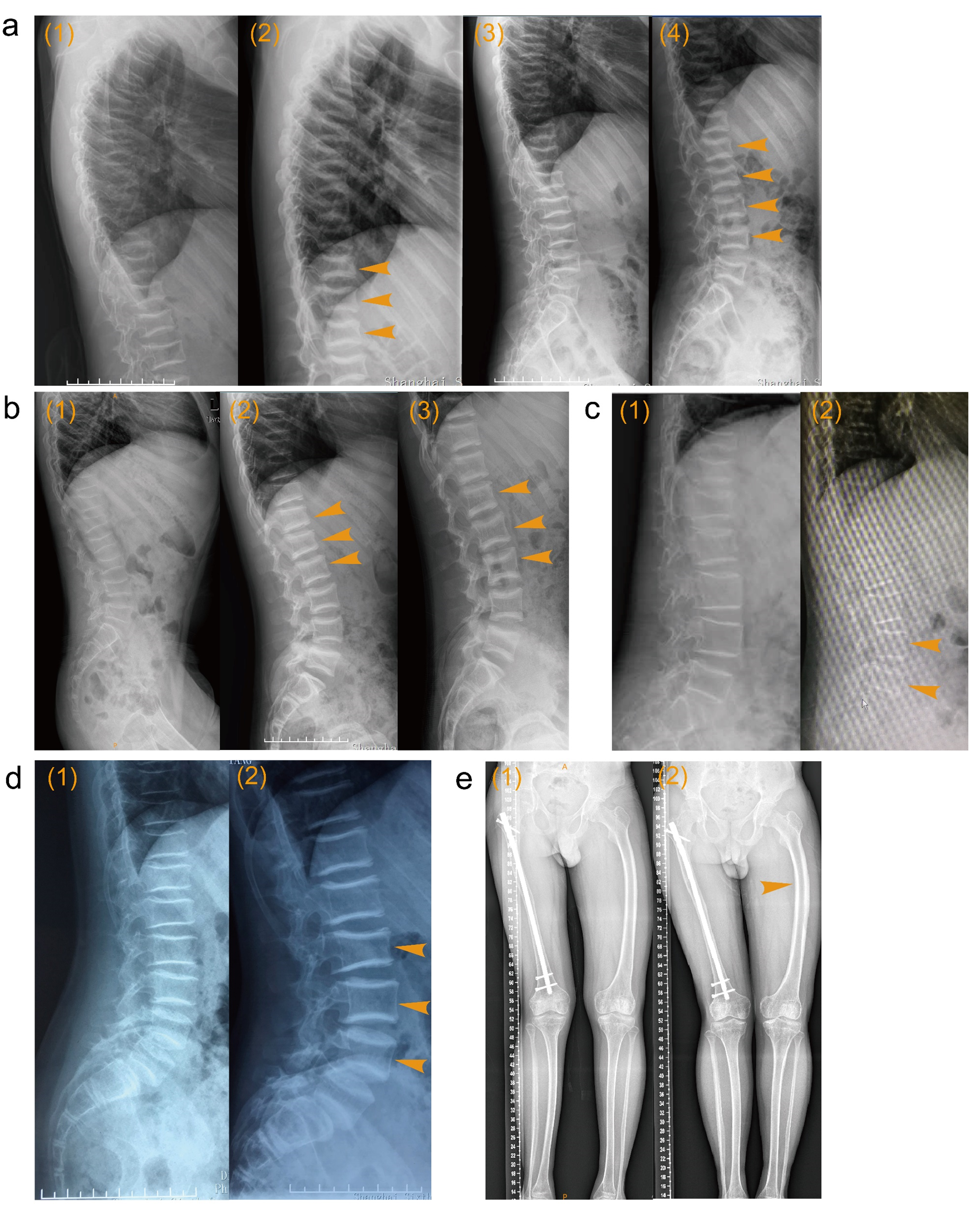

Supplement: Supplementary Figure 2 — Radiographic evidence of vertebral remodeling. (A) Thoracolumbar series (13M): Pre-treatment vs 12-month DEN. (B) Lumbar progression (13.9M): Baseline to 4-year ALN to 1-year DEN. (C) 6-month ALN effects (12.7M). (D) 12-month ALN outcomes (10.9M). (E) Lower extremity cortical changes (15M): 24-month ALN comparison. Orange arrows denote vertebral remodeling sites/cortical thickening. [file Image2.tif]
